# The effect of a high-impact jumping intervention on bone mass, bone stiffness and fitness parameters in adolescent athletes

**DOI:** 10.1007/s11657-018-0543-4

**Published:** 2018-11-17

**Authors:** Dimitris Vlachopoulos, Alan R. Barker, Esther Ubago-Guisado, Craig A. Williams, Luis Gracia-Marco

**Affiliations:** 1 Children’s Health and Exercise Research Centre, Sport and Health Sciences, College of Life and Environmental Sciences, University of Exeter, St. Luke’s Campus, Exeter, EX1 2LU UK; 2 IGOID Research Group, University of Castilla-La Mancha, Toledo, Spain; 3 Growth, Exercise, Nutrition and Development Research Group, University of Zaragoza, Zaragoza, Spain; 4 PROFITH “PROmoting FITness and Health through physical activity” Research Group, Department of Physical Education and Sports, Faculty of Sport Sciences, University of Granada, Granada, Spain

**Keywords:** Adolescence, Bone mass, Plyometric jump training, Football, Cycling, Swimming

## Abstract

***Summary*:**

This study demonstrates that a 9-month jumping intervention can improve bone mass gains and physical fitness performance in adolescent males participating in non-osteogenic sports, such as swimming and cycling.

**Purpose:**

To examine the effect of a jumping intervention on bone mass, bone stiffness and fitness parameters in adolescents involved in different sports.

**Methods:**

Ninety-three adolescent male swimmers (SWI), footballers (FOO) and cyclists (CYC) were randomised to intervention (INT) and sport (INT-SWI = 19, INT-FOO = 15, INT-CYC = 14) or sport only (CON-SWI = 18, CON-FOO = 15, CON-CYC = 12) groups. The 9-month jumping intervention consisted of 3 levels (12 weeks each) of 20 repetitions per set of counter movement jumps (CMJ) using adjustable weight vests (level 1 = 20 CMJ jumps/set, 0 kg, 3 sets/day, 3 times/week; level 2 = 20 CMJ jumps/set, 2 kg, 4 sets/day, 3 times/week; level 3 = 20 CMJ jumps/set, 5 kg, 4 sets/day, 4 times/week). Total body bone mineral content (BMC) at total body less head (TBLH) was measured using dual-energy X-ray absorptiometry and bone stiffness using quantitative ultrasound. Fitness was assessed using the 20-m shuttle run (20mSRT), CMJ and standing long jump (SLJ) tests.

**Results:**

INT-SWI had significantly higher increase in BMC legs and bone stiffness compared to CON-SWI (4.2–12.7%). INT-CYC had significantly higher increase in BMC at TBLH and legs and bone stiffness compared to CON-CYC (5.0–12.3%). There were no significant differences between INT-FOO and CON-FOO in any bone outcomes (0.9–3.9%). The increase in CMJ performance was significantly higher in INT-SWI (3.1 cm) and INT-CYC (3.2 cm) compared to CON-SWI and CON-CYC groups, respectively.

**Conclusions:**

A 9-month jumping intervention can improve bone mass, bone stiffness and muscular fitness in adolescent males participating in non-osteogenic sports, such as swimming and cycling.

**Clinical Trial Registration:**

ISRCTN17982776.

## Introduction

Adolescence is characterised by rapid changes in the skeletal system with 80–90% of total bone mass acquired by late adolescence depending on the skeletal site [1, 2]. The achievement of a high peak bone mass depends on genetic predisposition and environmental factors, such as the external loading applied on the skeleton via exercise during adolescence [3, 4]. Exercise can enhance bone mineral content (BMC) and bone mineral density (BMD) acquisition and the benefits can be maintained until adulthood [5, 6]. However, the bone gains are dependent on the loading characteristics of the sports practised [7, 8].

Participation in “osteogenic” sports, such us football, can augment BMC at loaded sites of the skeleton, such as the legs [9–11]. However, participation in “non-osteogenic sports”, such as swimming and cycling, may not benefit bone health [12, 13], which may affect bone development during adolescence and increase the risk of osteoporotic fractures in adulthood [14]. The total body less head (TBLH) BMC from dual-energy X-ray absorptiometry (DXA) has been considered one of the preferred methods of assessment for bone status in children and adolescents according to the International Society for Clinical Densitometry [15]. Additionally, quantitative ultrasound (QUS) can provide additional information regarding bone stiffness at the calcaneus site which is particularly important for adolescent athletes due to their high prevalence of injuries [16]. In a previous cross-sectional study, we found that adolescent male swimmers and cyclists had lower adjusted BMC at TBLH and legs and bone stiffness compared to footballers [17]. Furthermore, lean mass and fitness are important, significant positive predictors of bone outcomes in male youth athletes [18]. Adolescent footballers are thought to obtain the osteogenic stimulus needed to optimise their bone health through the sport-specific weight-bearing training [19]. In contrast, adolescent swimmers and cyclists may not obtain the optimal bone mineralisation during this critical period due to the lack of osteogenic stimulus [12, 13]. Recently, we have found that a 9-month jumping intervention improved bone mass (4.6–9.8%) and geometry (4.4–11%) outcomes at the lumbar spine and femoral neck in adolescent cyclists, and bone mass (6.0%) and geometry (10.9%) at the femoral neck in swimmers, but there were no improvements in footballers [20]. Therefore, a jumping intervention could improve bone stiffness at the ankles and bone mass at other skeletal sites, such as TBLH and legs of adolescent males involved in non-osteogenic sports.

In addition to skeletal benefits, fitness parameters may be improved following a jumping intervention [21]. Fitness tests, such as counter movement jumps (CMJ), standing long jumps (SLJ) and 20-m shuttle run test (20mSRT), are linked with sport-specific performance and can provide useful information regarding muscle strength, power and aerobic fitness levels, respectively [22, 23]. Jump power deriving from CMJ has been identified as a potential predictor of bone health in children [24], and previous evidence indicates that lower leg muscle power positively predicts bone strength in adolescent males and females [25]. The concept of muscle and bone as a functional unit is of particular importance for adolescent athletes considering the strong association between muscle and bone indices, and the increased muscle contractions and the impact applied on the skeleton during the sport-specific training [26]. Therefore, a jumping intervention may constitute an efficient strategy to improve fitness outcomes of adolescent athletes associated with bone health via the muscle–bone unit.

The purpose of the study was to examine the effects of a high-impact 9-month jumping intervention on bone mass, bone stiffness and fitness parameters in male adolescents involved in swimming, football and cycling. It was hypothesised that swimmers and cyclists, but not footballers will have improvements in bone mass, bone stiffness and jumping performance following the intervention programme.

## Methods

### Study design and participants

**Fig. 1:**
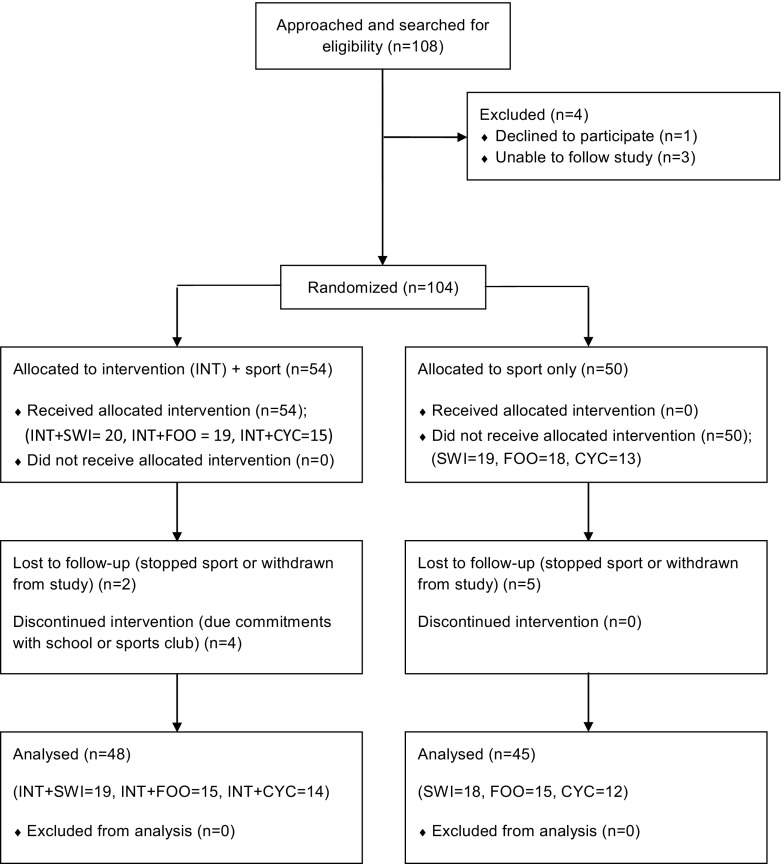
PRO-BONE study flow chart. CONSORT, Consolidated Standards of Reporting Trials

The present study represents a randomised controlled trial intervention as part of the longitudinal PRO-BONE study, whose methodology and previous findings has been described elsewhere [27–29]. The study design focused explicitly on the effect of the intervention on adjusted bone mass and stiffness of each specific sport (e.g. INT-SWI vs. CON-SWI) and not between sports. Inclusion criteria included the following: (1) Males 12–14 years old, engaged (≥ 3 h/week) in osteogenic (football) or non-osteogenic (swimming and cycling) sports in the last 3 years or more. Exclusion criteria included the following: (1) participation in another clinical trial; (2) any acute infection lasting until < 1 week before inclusion; (3) medical history of diseases or medications affecting bone metabolism or the presence of an injury (before inclusion) that may affect participation in their respective sports and/or any variable considered in the present study; (4) non-Caucasian participants. For the present study, data obtained at pre- (autumn/winter 2015/16) and post-intervention (summer/autumn 2016) programme (mean difference of visits = 289 days) were used. The Consolidated Standards of Reporting Trials (CONSORT) flow diagram is presented in Fig. 1. A total of 93 adolescent male athletes (14.1 ± 1.0 years) completed pre- and post-intervention. Each sport group was randomised by an independent researcher into two different groups: INT and sport (INT-SWI = 19, INT-FOO = 15, INT-CYC = 14) and sport only (without any additional intervention) (CON-SWI = 18, CON-FOO = 15, CON-CYC = 12).

### PRO-BONE study jump intervention programme

The 9-month progressive jump intervention programme (~ 10 min/day) consisted of CMJ and was performed by participants in the INT groups. The intervention consisted of 20 CMJ per set and 3 levels (12 weeks each) using adjustable weight vests (The Sports HQ, UK), and was performed on a hard surface. The intensity and the volume increased progressively by modifying the weight in the vests and the number of sets performed at each level (level 1 = 20 CMJ jumps per set, 0-kg weighted vest, 3 sets/day, 3 times/week; level 2 = 20 CMJ jumps per set, 2-kg weighted vest, 4 sets/day, 3 times/week; level 3 = 20 CMJ jumps per set, 5-kg weighted vest, 4 sets/day, 4 times/week). The intervention compliance was checked using diaries that were completed weekly by participants and parents by recording the number of jumps performed. The diary was returned to the research group every 3 months. The jumping intervention consisted of CMJ performed at a standard countermovement depth (90°) and arm-swing was not allowed. The participants completed the jumps continuously and landed with the knees bended to allow preparation for the next CMJ. Trained research assistants explained and demonstrated the CMJ only to participants in the INT groups, ensuring that the correct technique was used and that each participant could correctly execute a set of 20 CMJ before leaving the laboratory. The CMJ was chosen for the intervention as it has a high rate of change in force (493 times body weight/s) and ground reaction forces (5 times body weight) in 8.3–11.7-year-old boys and girls [30]. The reliability and validity of the CMJ has been previously reported [31].

### Dual-energy X-ray absorptiometry

A Lunar Prodigy DXA scanner (GE Healthcare Inc., WI, USA) was used to measure BMC (g), fat mass (g) and lean mass (g). The total body scan was used to obtain BMC at the arms (as a non-loaded site), legs and TBLH. All scans were undertaken by the same fully trained operator and the TBLH BMC was used as the main DXA outcome which is in line with the position of the International Society for Clinical Densitometry for DXA interpretation and reporting in children and adolescents [15]. The DXA percentage coefficient of variation has been reported between 1.0 and 2.9% at TBLH [32].

### Quantitative ultrasound

QUS measurements were performed with a Lunar Achilles Insight (TM Insight GE Healthcare, Milwaukee, WI, USA). This portable device measured bone stiffness using ultrasound waves, and measurements were always taken following manufacturer guidelines and by trained staff. QUS is considered a reliable, valid and radiation-free method to assess bone health in children [33].

### Anthropometry and maturity status

Height (cm) and body mass (kg) were measured by using standard procedures [27]. Somatic maturity status was assessed as years from peak height velocity using age and height in a validated algorithm (*R*^2^ = 0.90; standard error = 0.5) [34, 35].

### Physical activity, training characteristics and diet

Physical activity was measured for seven consecutive days at pre- and post-intervention using wrist accelerometers (GENEA, Cambridgeshire, UK). The validity and reliability of the accelerometer has been established previously in children and adolescents [36]. Data were collected at 100 Hz and analysed using 1-s epochs to establish time spent in moderate to vigorous physical activity (MVPA) using a validated cut point [36]. Weekly training hours were obtained by face to face interviews at pre- and post-intervention.

Total energy, protein and calcium intakes were assessed using a 24-h food recall. The validity and reliability of self-reported dietary intake has been previously reported in children [37]. Total energy, calcium and protein intake were estimated using the CompEat Pro software (Nutrition systems, VIS Visual Information Systems Ltd., UK).

### Cardiorespiratory and muscular fitness

Cardiorespiratory fitness was estimated using the 20mSRT [23] completed in the same sports hall as both pre- and post-intervention. The participants were encouraged to continue the test until they reached maximal effort. The test terminated when the participant failed to reach the line two consecutive times. The last completed shuttle determined the score of the test, and the number of shuttles completed was taken as an indicator of cardiorespiratory fitness. The 20mSRT has been shown to be reliable and valid in adolescents [38].

Muscular fitness was assessed using the SLJ test and the CMJ test at least 30 min before performing the 20mSRT and following a standardised warm up. For the SLJ, participants were advised to jump as far as possible in order to land with both feet and the distance (cm) measured between the starting line and the participant’s heels was recorded. The CMJ was assessed on a jump mat (Probotics Inc., AL, USA) which calculates jump height based on flight time and has established reliability and validity in adolescents [39]. For both CMJ and SLJ tests, three maximal jumps were performed and the best score was used. For countermovement jumps using the jump mat, the manufacturers reported an error of ± 0.001 s for flight time and ± 2.0 mm for jump height [40], and a validity study has previously reported that the relative error for jumping height was 3.5% (± 2.9%) [41].

### Statistical analyses

Statistical analyses were performed using the SPSS version 21.0 for Windows (IBM statistics, Chicago, IL, USA). Data were checked for normality and presented as mean and standard deviation. Data were analysed for each sport group separately using (1) paired *t* tests and one-way analysis of variance to detect the differences in characteristics and fitness parameters between intervention and control groups of each sport at pre- and post-intervention, and (2) a one-way analysis of covariance (ANCOVA) with Bonferroni post hoc and controlling for baseline bone status, change in lean mass and POST-intervention maturity status (years from peak height velocity), to detect differences in 9-month adjusted gains (Δ BMC and Δ bone stiffness) between the intervention and control groups of each sport (e.g. INT-SWI vs CON-SWI). The selection of the covariates was based on relevant predictors of bone outcomes in adolescents [18, 42]. Percentages of difference between the intervention and non-intervention groups were used to quantify the magnitude of the differences in adjusted bone outcome gains. Significance was set at *p* < 0.05.

## Results

### Descriptive characteristics

Details about the plyometric training programme and the compliance of the intervention are shown in Table 1. During the 9-month progressive jumping intervention, adverse events were reported from participants including soreness in the lower leg muscle groups (*n* = 8), pain in the knees mainly during the last stage of the intervention (*n* = 4) and fatigue (*n* = 6). The fatigue might explain the decline in the number of jumps completed in the final 12 weeks of the intervention. However, no intervention-related injuries were reported. Table 2 presents the descriptive characteristics of the participants pre- and post-intervention. No differences were observed in the descriptive characteristics presented in Table 2 between INT and CON groups at pre- and post-intervention for each specific sport, *p* > 0.05. In all INT groups, the studied variables significantly increased from pre- to post-intervention, except MVPA and fat mass in INT-CYC. Similarly, all variables significantly increased from pre- to post-intervention in CON-SWI, CON-FOO and CON-CYC except MVPA, and fat mass. The change in body mass, lean mass and fat mass did not differ between INT and CON groups of each sport. Total energy, protein and calcium intakes did not change pre- and post-intervention for any of the groups.

**Table 1:** PRO-BONE study plyometric jump intervention training progression and compliance

Level	Exercise	Vest weights (kg)	Repetitions	^2^Sets/day (^3^Rest)	^4^Trainings/week	Jumps/week	^5^Compliance in % and number (SD) of jumps completed		
							INT-SWI	INT-FOO	INT-CYC
1	^1^CMJ	–	20	3	3	180	90.3%	95.3%	91.3%
Total level 1 (12 weeks)					180 × 12 =	2160	1949 (204)	2059 (155)	1971 (240)
2	^1^CMJ	2	20	4	3	240	75.0%	83.9%	83.1%
Total level 2 (12 weeks)					240 × 12 =	2880	2159 (434)	2416 (444)	2393 (454)
3	^1^CMJ	5	20	4	4	320	46.0%	56.8%	47.1%
Total level 3 (12 weeks)					320 × 12 =	3840	1765 (298)	2181 (434)	1807 (598)
Total intervention (36 weeks)						8880	66.0% 5858 (1051)	75.0% 6656 (1281)	69.5% 6171 (1097)

^1^Countermovement jump

^2^Sets = 20 counter movement jumps

^**3**^Rest between sets = 30 s

^4^When 3 sets/day, jumps suggested to be performed in the morning before going to school (1 set), after school (1 set) and before going to bed (1 set). When 4 sets/day, jumps performed in the morning before going to school (1 set), after school (2 sets) and before going to bed (1st set)

^5^No significant differences between the intervention groups at any level of the intervention

**Table 2:** Characteristics of the sports groups and the control group before (PRE) and after (POST) the 9-month intervention programme

	Swimmers				Footballers				Cyclists			
Total (*N* = 93)	Intervention (*N* = 19)		Control (*N* = 18)		Intervention (*N* = 15)		Control (*N* = 15)		Intervention (*N* = 14)		Control (*N* = 12)	
	PRE	POST	PRE	POST	PRE	POST	PRE	POST	PRE	POST	PRE	POST
Age (years)	14.5 ± 0.9	*15.3 ± 0.9*	14.7 ± 1.1	*15.4 ± 1.1*	13.8 ± 1.0	*14.6 ± 1.0*	13.7 ± 0.8	*14.5 ± 0.8*	14.1 ± 1.1	*14.9 ± 1.1*	14.1 ± 0.9	*14.9 ± 0.9*
Height (cm)	170.3 ± 10.0	*174.1 ± 9.6*	172.8 ± 7.6	*176.0 ± 6.9*	160.5 ± 11.0	*165.4 ± 11.1*	163.3 ± 9.3	*168.9 ± 9.1*	168.2 ± 10.7	*174.5 ± 8.2*	162.7 ± 9.7	*166.4 ± 9.6*
Body mass (kg)	57.2 ± 9.0	*62.6 ± 7.8*	60.6 ± 7.2	*63.6 ± 7.7*	49.3 ± 10.8	*54.8 ± 11.6*	49.6 ± 7.4	*55.4 ± 7.1*	57.7 ± 13.0	*62.6 ± 12.2*	51.0 ± 12.4	*55.2 ± 13.9*
Lean mass (kg)	46.7 ± 9.4	*51.2 ± 8.5*	48.7 ± 8.2	*51.9 ± 6.7*	41.5 ± 10.0	*46.1 ± 10.5*	39.6 ± 7.4	*44.9 ± 7.2*	45.0 ± 7.9	*49.5 ± 7.6*	40.3 ± 8.6	*44.0 ± 9.2*
Fat mass (kg)	7.6 ± 3.1	*8.5 ± 2.9*	8.2 ± 3.4	8.7 ± 3.8	5.4 ± 2.2	*6.1 ± 2.3*	7.1 ± 2.3	7.6 ± 3.5	9.9 ± 9.7	10.0 ± 9.7	8.1 ± 5.5	8.3 ± 6.5
Years from PHV	1.0 ± 1.0	*1.7 ± 1.0*	1.2 ± 1.0	*1.8 ± 0.9*	0.0 ± 1.0	*0.7 ± 1.0*	0.1 ± 0.8	*0.9 ± 0.8*	0.7 ± 1.1	*1.4 ± 1.0*	0.3 ± 0.9	*1.0 ± 1.0*
Maturation (I/II/III/IV/V) (%)	(10/10/10/50/20)	*(0/0/11 /52/37)*	(0/11/16 /47/26)	*(0/0/6 /61/33)*	(10/10/43/37/0)	*(0/13/13 /40/34)*	(0/22/28 /50/0)	*(0/7/27 /40/27)*	(7/7/20 /53/13)	*(0/0/14 /43/43)*	(8/15/8 /61/8)	*(0/8/17 /42/33)*
MVPA (min/day)	61.3 ± 19.6	67.5 ± 19.6	59.6 ± 25.2	60.0 ± 18.1	97.7 ± 18.8	83.3 ± 18.5	89.6 ± 33.5	76.3 ± 22.4	85.6 ± 22.1	93.9 ± 15.7	88.9 ± 21.8	78.7 ± 13.4
Training volume (h/week)	7.9 ± 3.6	*11.8 ± 5.4*	10.2 ± 3.1	*12.9 ± 5.4*	10.2 ± 1.2	*12.0 ± 2.8*	8.8 ± 2.0	*10.7 ± 1.8*	5.3 ± 2.0	*7.9 ± 3.8*	5.3 ± 1.6	*8.2 ± 2.8*
Energy intake (kcal/day)	2534 ± 382	2465 ± 221	2603 ± 425	2386 ± 133	2237 ± 517	2379 ± 262	2419 ± 620	2309 ± 234	2320 ± 280	2244 ± 166	2221 ± 325	2226 ± 152
Protein intake (g/day)	85.8 ± 30.9	89.8 ± 31.2	77.9 ± 28.5	82.8 ± 24.8	95.3 ± 21.6	97.1 ± 29.3	92.0 ± 35.3	90.1 ± 20.4	80.3 ± 13.3	88.0 ± 23.0	84.9 ± 28.9	86.4 ± 21.1
Calcium intake (mg/day)	1237 ± 280	1118 ± 289	1155 ± 257	1109 ± 189	1342 ± 350	1231 ± 167	1177 ± 378	1179 ± 178	1183 ± 370	1253 ± 196	1385 ± 280	1279 ± 149

Values are mean ± standard deviation. No differences observed at PRE and POST between INT and CON groups of each specific sport, *p* > 0.05. Italicized values denote significant different values between PRE and POST, *p* < 0.05

*MVPA*, moderate to vigorous physical activity; *PHV*, peak height velocity

### Bone mass and bone stiffness

**Fig. 2:**
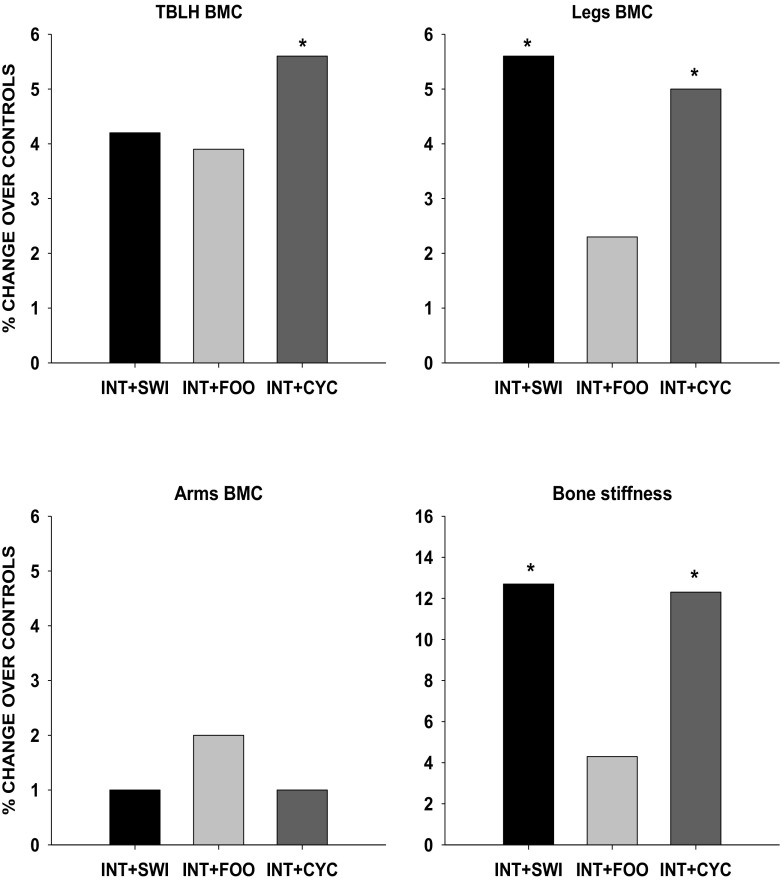
The effect of 9-month jumping intervention on adjusted change in bone mineral content (BMC, g) and bone stiffness presented as percentage (%) over control groups (0 lines). Results were adjusted for baseline (pre) bone outcomes, change in lean mass and post maturity status. Asterisk denotes significant higher change compared to the sport specific control group, *p* < 0.05

Table 3 shows PRE-intervention BMC (g) and the 9-month adjusted gains in BMC (g) and bone stiffness in all groups. Figure 2 shows the percentage of difference on adjusted BMC and bone stiffness gains between each of the INT-SPORT and CON-SPORT groups. No differences were observed in bone outcomes at PRE-intervention between INT and CON groups of each specific sport, *p* > 0.05. INT-SWI gained significantly higher leg BMC (5.6%, *p* = 0.011) and bone stiffness (12.6%, *p* = 0.001) than CON-SWI. There were no significant differences in INT-SWI and CON-SWI for TBLH BMC and for arm BMC (1.0–4.1%, *p* > 0.05). INT-CYC gained significantly higher TBLH BMC (5.6%, *p* = 0.014), leg BMC (5.0%, *p* = 0.002) and bone stiffness (12.3%, *p* = 0.001) than CON-CYC. There were no significant differences in INT-CYC and CON-CYC for arm BMC (1.0%, *p* > 0.05). There were no significant differences between INT-FOO and CON-FOO (0.9–3.9%, *p* > 0.05) for the bone outcomes.

**Table 3:** PRE and 9-month adjusted gain in bone mineral content (BMC, g) and bone stiffness of the intervention and control groups

Total (*N* = 93)	Swimmers				Footballers				Cyclists			
	Intervention (*N* = 19)		Control (*N* = 18)		Intervention (*N* = 15)		Control (*N* = 15)		Intervention (*N* = 14)		Control (*N* = 12)	
	PRE	9-month adjusted gains Δ (95% CI)	PRE	9-month adjusted gains Δ (95% CI)	PRE	9-month adjusted gains Δ (95% CI)	PRE	9-month adjusted gains Δ (95% CI)	PRE	9-month adjusted gains Δ (95% CI)	PRE	9-month adjusted gains Δ (95% CI)
TBLH BMC	1892 ± 339	322 (276–368)	1952 ± 325	251 (203–300)	1730 ± 479	386 (335–438)	1769 ± 398	325 (274–377)	1848 ± 379	*341 (290–394)*	1582 ± 401	203 (145–260)
Leg BMC	888 ± 147	*141 (124–159)*	922 ± 135	95 (77–114)	877 ± 246	150 (130–170)	886 ± 201	131 (111–150)	899 ± 170	*148 (129–168)*	759 ± 168	87 (65–109)
Arm BMC	290 ± 68	44 (37–51)	303 ± 67	40 (33–47)	226 ± 76	46 (38–53)	228 ± 61	48 (41–56)	271 ± 68	49 (40–56)	233 ± 74	40 (32–48)
Bone stiffness	95.9 ± 12.0	*10.3 (7.2–13.5)*	94.5 ± 16.3	−1.8 (−5.1–1.6)	104.4 ± 11.9	15.3 (11.6–8.9)	105.7 ± 12.4	10.9 (7.2–14.6)	95.6 ± 15.6	*9.5 (5.9–13.1)*	93.6 ± 14	−2.2 (−6.0–1.7)

Raw values at PRE are mean ± standard deviation. Values at 9-month were adjusted for pre bone values, change in lean mass and post peak height velocity, and presented as mean and 95% CI. No differences observed in bone outcomes at PRE between INT and CON groups of each specific sport, *p* > 0.05. Italicized values denote significant higher adjusted bone gains between the intervention and control group of each specific sport, *p* < 0.05

*BMC*, bone mineral content; *TBLH*, total body less head

### Physical fitness parameters

Table 4 presents physical fitness measurements of the groups pre- to post-intervention. No differences were observed in fitness parameters at pre-intervention between INT and CON groups of each specific sport, *p* > 0.05. The 9-month change in CMJ of INT-SWI (3.1 cm) and INT-CYC (3.2 cm) was significantly higher compared to that of CON-SWI (− 0.6 cm) and CON-CYC (0.7) respectively, *p* < 0.05. The 9-month change in SJ and 20mSRT was not significantly different between INT and CON groups. INT-SWI and INT-CYC significantly increased CMJ (6.4–7.2%), SLJ (3.7–4.8%) and 20mSRT (7.0–7.9%) from pre- to post-intervention (all *p* < 0.05). In INT-FOO, CMJ (4.7%) and SLJ (4.0%) were significantly increased from pre- to post-intervention (all *p* < 0.05), but 20mSRT did not (3.9%). In CON-SWI and CON-CYC, none of the fitness parameters (1.5–2.5%) improved (*p* > 0.05). CON-FOO significantly increased CMJ (4.0%) from pre- to post-intervention (*p* < 0.05).

**Table 4:** Physical fitness measurements of the sports groups and the control group before (PRE) and after (POST) the 9-month intervention programme

	Swimmers				Footballers				Cyclists			
Total (*N* = 93)	Intervention (*N* = 19)		Control (*N* = 18)		Intervention (*N* = 15)		Control (*N* = 15)		Intervention (*N* = 14)		Control (*N* = 12)	
	PRE	9-month change	PRE	9-month change	PRE	9-month change	PRE	9-month change	PRE	9-month change	PRE	9-month change
Counter movement jump (cm)	46.8 ± 7.2	3.1 ± 0.4*	46.5 ± 9.4	−0.6 ± 0.3	45.3 ± 6.1	*2.2 ± 0.3*	41.7 ± 5.8	*1.7 ± 0.5*	42.7 ± 5.9	3.2 ± 0.6*	45.4 ± 7.8	0.7 ± 0.4
Standing long jump (cm)	195.8 ± 27.8	*7.3 ± 1.8*	191.1 ± 27.7	3.6 ± 2.0	188.1 ± 24.9	*6.5 ± 2.6*	184.1 ± 21.2	7.6 ± 3.8	173.8 ± 34.1	*9.1 ± 2.2*	180.7 ± 24.5	4.6 ± 3.6
20mSRT (shuttles)	79.2 ± 17.6	*6.5 ± 1.7*	74.2 ± 24.4	1.9 ± 1.4	94.5 ± 14.4	3.8 ± 1.3	92.0 ± 21.9	2.9 ± 1.6	82.4 ± 24.2	*6.1 ± 1.0*	82.8 ± 19.2	2.8 ± 1.6

Values are mean ± standard deviation. No differences observed at pre between INT and CON groups of each specific sport, *p* > 0.05. Italicized values denote a significant increase between pre and post, *p* < 0.05

*20mSRT*, 20-m shuttle run test

*A significant higher increase in the INT group compared to the CON group of the specific sport, *p* < 0.05

## Discussion

The main findings of the present study were that a high-impact jumping intervention can (1) induce significant improvements in TBLH BMC, leg BMC and bone stiffness in athletes engaged in the non-osteogenic sports, swimming and cycling, but not in the osteogenic sport, football; and (2) induce significant improvements in muscular fitness in non-osteogenic sports groups. Collectively, this study shows that a progressive jump training programme can be implemented by sports clubs to improve bone mass, bone stiffness and muscular fitness in male adolescent athletes who participate in non-osteogenic sports such as swimming and cycling.

### Effectiveness of the PRO-BONE jumping intervention on bone mass and bone stiffness

The effect of the PRO-BONE jumping intervention on bone stiffness in non-osteogenic groups was greater in magnitude compared to the effects to bone mass in previous jumping intervention studies in children and adolescents [43–45]. A previous 8-month school-based jumping intervention reported that non-athletic adolescent males had 4.3% significantly higher gains at total body BMC and 5.0% improvements at QUS outcome compared to controls [43]. In the present study, the improvements in bone stiffness of both non-osteogenic sports were greater than the improvements in BMC at TBLH and legs. These greater site-specific improvements observed at the bone stiffness of the ankle may be explained by the higher ground reaction forces and stimulus applied on this skeletal site during the jumping intervention [12, 46]. We have previously showed that the BMC improvements were consistent with the improvements in bone turnover response as bone formation and resorption significantly decreased in CON-SWI and CON-CYC, but not in INT-SWI and INT-CYC following the intervention, indicating a potential protective effect of the jumping intervention against the reduction of bone turnover markers [20]. The bone response to the intervention could be a direct effect on bone and/or indirect through an augmentation of lean mass [47], and we have found that lean mass has the highest positive predictive role for bone outcomes in male adolescent athletes [18]. Previous 7- and 8-month jumping intervention studies in a school-based environment on non-athletic pubertal children and adolescents reported significant improvements on BMC (1.4–4.5%) in the intervention groups compared to controls [44, 45, 48]. In these previous studies, the effect of the intervention was greater at the weight-bearing sites of the skeleton (4.5%), which is equivalent with the higher improvements observed at leg BMC (5.0–5.6%) in the present study. In addition, the greater bone adaptations in the non-osteogenic groups of the current study compared to those in previous work may be attributed to the ability of the skeleton to adapt better to the external stimulus after long-term (8 and 6 years for swimmers and cyclists, respectively) non-osteogenic sport participation [10]. In contrast, but consistent with our hypothesis, the stimulus provided by the jumping intervention was not enough to induce significant bone gains in INT-FOO compared to CON-FOO, despite footballers showing better compliance to the intervention compared to cyclist and swimmers (75% vs 69.5% and 66%, respectively, *p* > 0.05). Compliance to the jumping programme in the present study was slightly lower than that to other studies (70% vs 80–90%) [48, 49], and the decline observed in the last 12 weeks might be due to the longer duration (9 months vs 7–8 months) and/or due to the greater number of jumps performed in the present study (160 vs 90 jumps per week) [44]. According to the mechanostat theory, the bones adapt their strength and content to respond to the strain caused by external physiological loads up to a certain point [50, 51]. The lack of significant effect of the intervention in footballers might be explained by the osteogenic stimulus that footballers already receive from the participation in football. Footballers may have reached a ceiling for bone improvements as we have previously shown to have greater bone outcomes compared to swimmers and cyclists after adjusting for lean mass among other confounders [17]. The implemented jumping intervention significantly improved bone outcomes in non-osteogenic sports, such as swimming and cycling, compared to their respective control groups indicating an opportunity to counteract the lack of osteogenic stimulus observed in adolescent athletes involved in non-osteogenic sports [12].

### Effectiveness of the PRO-BONE jumping intervention on physical fitness parameters

Physical fitness parameters, and specifically jumping performance, have been considered strong markers of bone health and are associated with lean mass and bone development during adolescence [24, 52]. The present intervention induced significant improvements in muscular fitness derived from CMJ in non-osteogenic INT groups. INT-SWI (3.1 cm) and INT-CYC (3.2 cm) improved CMJ compared to CON-SWI (− 0.6 cm) and CON-CYC (0.7 cm), respectively. However, INT-FOO did not improve CMJ which might be explained by our previous cross-sectional findings showing that footballers already had a higher physical fitness performance in CMJ compared to swimmers and cyclists [53]. Therefore, the potential to improve CMJ performance from the present jumping intervention programme is greater in swimmers and cyclists than that in footballers. Additionally, 20mSRT significantly improved (7.0–7.9%) in INT-SWI and INT-CYC, but not in INT-FOO (3.9%). Improvements in 20mSRT performance partially depend on muscle power, which is a combination of force and velocity. Lean mass is the main determinant of force in adolescent athletes, and it is related to BMC and bone stiffness [18]. Part of the improvements in BMC and stiffness, and the performance in the 20mSRT, could be explained through the strong association of lean mass to both bone outcomes and the 20mSRT [54]. While the lack of improvement in the 20mSRT in footballers could be explained by their already high level of aerobic fitness, according to the “Functional Muscle-Bone-Unit”, the largest physiological loads are caused by muscle contractions due to the greater muscle forces produced during participation in weight-bearing activities [55]. The improvements observed in bone outcomes and muscle function in the present study might be related to the progressive weight-bearing loading applied to the lower leg muscle groups during the jumping intervention programme [56]. The findings of the present study are in accordance with a 10-month intervention programme that included small-sided ball games and circuit strength training groups and found 9–10% higher improvements on CMJ distance of both non-athletic 8–10-year-olds compared to controls [57]. The magnitude of improvement in muscular fitness in the present study might be explained by the dose–response of benefits of plyometric training on physical performance in adolescent athletes [58]. The movement characteristics of football already include mechanical forces applied on the skeleton that are generated either through ground impact or muscle contractions which might indicate a better muscle–bone unit function and explain lack of further improvement in CMJ following the intervention [59]. Additionally, based on the inclusion criteria, all sports groups had undertaken > 3 h per week of specific training for the past 3 years at baseline. The footballers reported higher levels of participation in plyometric training (INT-FOO = 57%, CON-FOO = 55%) compared to cyclists (INT-CYC = 29%, CON-CYC = 26%) and swimmers (INT-SWI = 43%, CON-SWI = 41%), which may account for the significant fitness adaptations in the INT-SWI and INT-CYC groups.

### Strengths and limitations

The strengths of the present study include (1) the investigation of a high-impact jumping intervention on bone mass and bone stiffness; and (2) the effects of a high-impact jumping intervention on muscular fitness in adolescent athletes participating in different loading sports. The limitations of the study should also be noted. DXA is a clinically relevant device to assess bone outcomes, but the two-dimensional imaging technique cannot provide information regarding structural adaptations that may be induced from the jumping intervention. A limitation of the present study is the relatively small sample size in each group, but the study was adequately powered based on sample size calculations [27, 60]. The jump mats use flight time to calculate jump height, thus caution should be given to the method of calculation used. Nevertheless, standardisation was followed and trained researchers ensured participants performed the jumps appropriately and as instructed.

## Conclusion

The present study indicates that a 9-month high-impact jumping intervention can significantly improve BMC at TBLH and legs, and bone stiffness in adolescent male athletes involved in non-osteogenic sports, such as swimming and cycling. The intervention also induced significant improvements in muscular fitness of non-osteogenic sport groups. The intervention programme could be implemented by non-osteogenic sports clubs and athletes to improve bone mass, stiffness and physical fitness.

## Abbreviations

BMC: Bone mineral content
BMD: Bone mineral density
CMJ: Counter movement jump
CON: Controls
CYC: Cyclists
DXA: Dual-energy X-Ray absorptiometry
FOO: Footballers
INT: Intervention
MVPA: Moderate to vigorous physical activity
QUS: Quantitative ultrasound
SLJ: Standing long jump
SWI: Swimmers
TBLH: Total body less head
